# Examining for an association between candidate gene polymorphisms in the metabolic syndrome components on excess weight and adiposity measures in youth: a cross-sectional study

**DOI:** 10.1186/s12263-017-0567-1

**Published:** 2017-07-04

**Authors:** Angélica María Muñoz, Claudia María Velásquez, Gloria María Agudelo, Rosa Magdalena Uscátegui, Alejandro Estrada, Fredy Alonso Patiño, Beatriz Elena Parra, María Victoria Parra, Gabriel Bedoya

**Affiliations:** 1 Research Group on Food and Human Nutrition, Universidad de Antioquia (UdeA), Calle 70 No. 52-21, Medellin, Colombia; 2 Vidarium Research Group, Nutrition, Health and Wellness Research Center, Nutresa Business Group (Grupo Empresarial Nutresa), Calle 8 Sur No. 50-67, Medellin, Colombia; 3 Universidad de Antioquia (UdeA), Calle 70 No. 52-21, Medellin, Colombia; 4 Research Group on Demography and Health, Universidad de Antioquia (UdeA), Calle 70 No. 52-21, Medellin, Colombia; 5 Research Group of Sciences Applied to Physical Activity and Sports, Universidad de Antioquia (UdeA), Calle 70 No. 52-21, Medellin, Colombia; 6 Molecular Genetics Group, Universidad de Antioquia (UdeA), Calle 70 No. 52-21, Medellin, Colombia; 7 Sede de Investigación Universitaria (SIU), Universidad de Antioquia (UdeA), Calle 62 No. 52-59, Laboratorio 413, Medellin, Colombia

**Keywords:** Body mass index, Adiposity, Polymorphisms, Environmental factors, Youth

## Abstract

**Background:**

A polymorphism in a gene may exert its effects on multiple phenotypes. The aim of this study is to explore the association of 10 metabolic syndrome candidate genes with excess weight and adiposity and evaluate the effect of perinatal and socioeconomic factors on these associations.

**Methods:**

The anthropometry, socioeconomic and perinatal conditions and 10 polymorphisms were evaluated in 1081 young people between 10 and 18 years old. Genotypic associations were calculated using logistic and linear models adjusted by age, gender, and pubertal maturation, and a genetic risk score (GRS) was calculated by summing the number of effect alleles.

**Results:**

We found that AGT-rs699 and the IRS2-rs1805097 variants were significantly associated with excess weight, OR = 1.25 (CI 95% 1.01–1.54; p = 0.034); OR = 0.77 (CI 95% 0.62–0.96; p = 0.022), respectively. AGT-rs699 and FTO-rs17817449 variants were significantly and directly associated with body mass index (BMI) (p = 0.036 and p = 0.031), while IRS2-rs1805097 and UCP3-rs1800849 were significantly and negatively associated with BMI and waist circumference, correspondingly. Each additional effect allele in GRS was associated with an increase of 0.020 log(BMI) (p = 0.004). No effects from the socioeconomic and perinatal factors evaluated on the association of the candidate genes with the phenotypes were detected.

**Conclusions:**

Our observation suggests that AGT-rs699 and FTO-rs17817449 variants may contribute to the risk development of excess weight and an increase in the BMI, while IRS2-rs1805097 showed a protector effect; in addition, UCP3- rs1800849 showed a decreasing waist circumference. Socioeconomic and perinatal factors had no effect on the associations of the candidate gene.

**Electronic supplementary material:**

The online version of this article (doi:10.1186/s12263-017-0567-1) contains supplementary material, which is available to authorized users.

## Background

The increasingly early onset of overweight and its long-term consequences focus the need to develop interventions for children, teens, and young adults [1]. Obesity, especially the central type, generates conditions that increase the risk of metabolic syndrome (MetS), defined as a set of traits that increase the risk of cardiovascular disease and diabetes mellitus 2 [2].

Being overweight is a result of a continuing imbalance between consumption and energy expenditure of an individual, where food consumption depends on, among others, environmental factors such as availability and genetic factors that influence appetite. Energy expenditure may also be affected by both lifestyle and metabolic efficiency, which, in turn, is influenced by genetic factors [3, 4].

Studies have shown that a polymorphism in a gene related to a given system may exert its effects in other pathways and influence multiple phenotypes [5]. Several loci susceptible to the components of MetS, such as those evaluated in this study, may play a role in the risk of excess weight. Uncoupling Protein-3 (*UCP3*) is involved in energy expenditure by stimulating thermogenesis, making it an attractive target for studies on the regulation of body weight [6]; Calpain 10 (*CAPN10*) is involved in numerous cellular functions, including signaling and adipocyte differentiation [7]; fat mass and obesity-associated (*FTO*) increases energy intake by regulating the expression of genes that control appetite [8]. The Insulin Receptor Substrate 2 (*IRS2*) and the protein that codes the transcription factor of the 7-like 2 (*TCF7L2*) gene play an important role in the transduction of insulin signaling [9, 10]; variants in the angiotensin-converting enzyme (*ACE*) and angiotensinogen (*AGT*) genes are associated with hypertension, finding the protein expression in adipose tissue [11]; in fact, variations in ATP-binding cassette, sub-family A (ABC1), member 1 (*ABCA1*), lipoprotein lipase (*LPL*), and cholesteryl ester transfer protein (*CETP*), associated with lipoprotein metabolism, contribute to the variation in adipogenesis.

Given these interactions between genetic and environmental factors in the etiology of obesity, increasing evidence reports differences in the frequency of these variants among populations, ethnicities, genders, and socioeconomic stratum [12, 13]. Studies related to the presence of polymorphisms associated with the development of adiposity measures have demonstrated discrepant results among population [14, 15]. Latin American populations, such as those in Colombia, are the result of a recent admixture among three ancestral populations: European, African, and Amerindian. Admixture can result in population stratification and may lead to spurious associations rather than association of genes with disease if the allele frequencies differ among the groups because of systematic differences in ancestry. In addition, socioeconomic stratum and parental education may be related to food availability and the transmission of a food culture [16, 17]; however, their relationship to the development of obesity is not clear enough. Results in a Mediterranean population suggest that education may modify the genetic susceptibility of *FTO* to obesity, with BMI being higher in non-university subjects compared to university subjects [18]. On the other hand, Pigeyre et al. [19] report the influence of maternal education in the association of neuromedin B rs3809508 and the risk of obesity. Perinatal conditions, such as increased or decreased fetal growth, as measured by weight and height at birth, or low breastfeeding, may also influence the likelihood of obesity later on in life [20].

Our hypothesis is that variants related to the MetS components may be associated with BMI and adiposity measures and this could be modified by socioeconomic and perinatal factors in young people aged 10 to 18 years.

## Methods

### Study design

The study is a cross-sectional study.

### Participants

The sample consisted of 1081 young people between 10 and 18 years of age from Colombia, selected by random sampling and who were participants of the cross-sectional study “Variations in the Prevalence of Metabolic Syndrome in Adolescents According to Different Criteria Used for Diagnosis” [21], a study conducted between 2011 and 2012. The sample size was calculated with a confidence level of 95%, with an estimated prevalence of overweight of 10.3% according to the National Nutritional Situation Survey (2005) [22], a sample error of 2%, and effect on design of 1.2.

Excluded from the study were those young people with a habitual consumption of medications, young people with diabetes and genetic diseases, who are highly competitive athletes, young pregnant women, or those who were breastfeeding.

### Measures

#### Perinatal and diseases history

The history of diseases (presence or absence of the disease) was considered in relatives to the second degree of consanguinity. Additionally, the perinatal history, such as birth weight (low <2500 g and high >4000 g), breastfeeding and duration, was also determined.

#### Parental education and socioeconomic stratum

Parental education and socioeconomic stratum were determined. The level of education was categorized into primary (0–6 years) and secondary (12 years and subsequent studies (>12 years); the stratum was determined as low (strata 1 and 2), medium (3 and 4), and high (5 and 6), according to the National Administrative Department of Statistics [23].

#### Pubertal maturation

The stage of pubertal maturation according to the methods established for this purpose [24, 25] was also evaluated.

#### Anthropometric evaluations

Weight, height, waist circumference, triceps, and subscapular fat fold were measured in all young people with equipment and international techniques before training and standardization of the evaluators; each measurement was assessed and recorded twice. For the classification of the nutritional status, the BMI (weight in kg/height in m^2^) was calculated; participants were classified according to the 2007 World Health Organization (WHO) BMI-for-age and gender reference standard [26]. Overweight was defined as BMI percentile ≥85.0th; adequate as BMI percentile <85.0th. Waist circumference was considered as being high with a value of more than p90 of the values for Mexican–American young people from the U.S. Third National Health and Nutrition Examination Survey [27]. The percentage of total body fat (%*BF*) was calculated with the subscapular and triceps fat folds, according to Lohman et al. [28] and was classified as obesity when it was >25% in males and >32% in females, adequate between 12 and 25% in males and 25 and 32% in females, and deficient when it was <12% in males and <15% in females [28].

#### Genotyping

After excluding individuals related up to the third degree of consanguinity determined through clinical histories and information provided in the General Information questionnaire, ten variants were genotyped in 1005 youth: *IRS2*-rs1805097, *CAPN10*-rs3842570, *UCP3*-rs1800849, *FTO*-rs17817449, *TCF7L2-*rs7903146, *AGT-*rs699, *ACE*-rs4340, *LPL*-rs285, CETP-rs708272, and *ABCA1*-rs2230806; the genotyping was performed by polymerase chain reaction-restriction fragment length polymorphism. The PCR was performed in a 25-μl volume containing 10 ng genomic DNA, 10 × PCR buffer with 1.5 mM MgCl_2_, 0.5 mM dNTPs, 0.5 units of Taq polymerase, and 5 μmol of each primer. PCR conditions included one step initial desaturation at 95 °C for 3 min, 35 cycles (95 °C for 45 s, 55–62 °C for 45 s, and 72 °C for 45 s), and a final extension at 10-min extension step at 72 °C. The PCR products were checked on 1.5% agarose gel. Amplified PCR products were digested with restriction enzyme overnight. The digestion products were electrophoresed on agarose gel and visualized by staining with ethidium bromide. Primers and restriction enzymes used are reported in the Additional file 1: Table S1. To assess reproducibility, 10% of the samples were doubly genotyped. Negative controls were also added to each 96-well plate. No discordance was detected between the replicated samples, and reproducibility was 98.9%.

#### Genetic ancestry estimation

Individual admixture proportions were available for 337 out of the 738 participants with normal weight, and for 235 of 267 subjects in the overweight group. For these individuals, European, Amerindian, and African contributions were estimated with the program ADMIXMAP v 3.2 [29], using a set of 40 ancestry informative markers (AIMs) broadly distributed across the genome and according to their significant differences in allele frequencies among two populations. These AIMs have been described previously to accurately estimate ancestry in Latin American populations [30] (Additional file 1: Table S2).

#### Statistical analysis

A descriptive analysis was performed on the perinatal history and the socioeconomic, health, anthropometric, and genetic variables. The comparison of the variables among the groups according to nutritional status with the Pearson *X* ^*2*^ and Pearson correlation was made; the Student *t* tests were made for quantitative variables. For the allele and genotype frequencies, the Hardy–Weinberg equilibrium and the associations of the variant phenotypes, PLINK v1.07 [31] were used. Logistic regression analysis was performed to look for associations of polymorphisms with nutritional status (normal weight and overweight groups) using the following models: additive (major allele homozygotes vs. heterozygotes vs. minor allele homozygotes), dominant (major allele homozygotes vs. heterozygotes + minor allele homozygotes), and recessive (major allele homozygotes + heterozygotes vs. minor allele homozygotes). The best model was chosen according to the Akaike information criterion. Logistic regression was employed to estimate the odds ratio (OR) and its confidence interval (CI) of 95%. The association of genotype with BMI, WC, and percentage of BF were evaluated using linear regression; variables with non-normal distributions were log-transformed before analysis. For the anthropometric measurements that were associated to more than two associated variants, an estimation of the individual genetic risk score (GRS) was generated, using the risk alleles. We quantified the unweighted genetic risk score to assess the combined effects of the variants by summing the number of risk alleles; each individual might have 0, 1, or 2 risk alleles in each of the variants. The weighted GRS was calculated by multiplying the number of risk alleles at each locus (0, 1, 2) by the variants’ β coefficient from the predictive model and then summing the product. Effects of covariables (gender, age, pubertal maturation, and BMI) were controlled, as well as the interaction between the genetic variants and the social stratum, maternal education year, birth weight, and maternal breastfeeding. In addition to the previous analysis, we conducted logistic analyses for the subset of the samples for which information ancestry was accessible. The objective of these analyses was to determine if the possible association of the polymorphism with nutritional status (normal weight and overweight groups) or adiposity measurement could be driven by population stratification. Controlling for two out of three admixture estimates avoided collinearity in the model, since the three ancestry components sum up to 1.

Multiple test correction was done via permutation tests; we performed 10,000 permutations to determine empirical significance, which was considered for a value of *p <* 0.05.

## Results

### Perinatal history and socioeconomic, health, and anthropometric conditions of the young people

Of the total of 1081 participants, 581 (52.7%) were females; the mean age was 14.2 ± 2.4 years. The prevalence of excess weight (percentile ≥85.0th) was 25.1% (272); of these, 13.7% (149) were overweight and 11.3% (123) had obesity (BMI percentile >95.0th).

No significant differences were found between the socioeconomic stratum and birth weight with the nutritional status; however, excess weight was significantly greater in young people with more-educated parents (*p <* 0.023), in those who had a family history of hypertension (*p =* 0.011), dyslipidemia (*p =* 0.003), and obesity (*p =* 0.00) and those who presented a minor duration of breastfeeding (*p =* 0.048) (see Table 1). Characteristics by gender are shown in Additional file 1: Table S3. There were no significant differences in socioeconomic status, parent education, family history of diseases, perinatal history, and BMI. The percentage of individuals who reported prepubertal maturation was significantly higher in men than in women (*p =* 0.011). In contrast, waist circumference and BF% was significantly higher in women than in men (*p <* 0.001 for both). A histogram of anthropometric variables, stratified by gender, is shown in Additional file 1: Figure S1

**Table 1:** Characteristics of the study population, stratified according to BMI

Variable	BMI <p 85.0th (%), *n =* 809	BMI ≥p 85.0th (%), *n =* 272	*p* value
Socioeconomic status^a^			
Low	43.3	39.3	0.082
Medium	37.8	36.4	
High	18.9	24.3	
Maternal education, year^a^			
0–6	15.3	7.7	**0.008**
+6–12	47.4	49.8	
+12	37.3	42.4	
Paternal education, year^a^			
0–6	14.8	10.6	**0.023**
+6–12	47.2	44.1	
+12	38.1	45.3	
Pubertal maturation^a^			
Prepubertal	18.2	18.4	0.155
Pubertal	26.6	33.8	
Postpubertal	55.3	47.8	
Family history^b^			
Obesity	34.4	60.1	**<0.001**
Type 2 diabetes	55.8	62.0	0.073
Gestational diabetes	2.7	2.6	0.897
Hypertension	76.0	83.4	**0.011**
Dyslipidemia	59.5	69.7	**0.003**
Birth weight, (g)^a^			
<2500	9.2	6.3	0.056
≥2500–4000	85.7	86.1	
>4000	5.1	7.5	
Maternal breastfeeding^b^	93.8	90.4	0.057
Duration of breastfeeding, (months)^a^			
0–1	6.2	9.7	**0.048**
>1–3	21.4	22.8	
>3–6	21.3	21.7	
>6	51.1	45.7	
Mean ± SD			
Anthropometry^c^			
Weight, kg	47.3 (11.0)	59.8 (15.0)	**<0.001**
BMI, kg/m^2^	19.0 (2.39)	24.4 (3.49)	**<0.001**
BF%	22,3 (7.8)	32.2 (8.11)	**<0.001**
Waist circumference, (cm)	65,0 (5.8)	76.2 (8.3)	**<0.001**

Data is shown as percentage (%) or average ± standard deviation The significant *p* values (*p <* 0.05) are given in bold

*BMI* body mass index, *BF%* body fat percentage

^a^Spearman correlation

^b^Pearson’s chi-square

^c^Student *t* test

.

### Association between excess weight and adiposity measurements with genetic variants

The genetic analysis considered those subjects unrelated, leaving a total of 1005 participants. Genotype distributions did not deviate from the Hardy–Weinberg expectations. An allelic frequency above 24% was found for the low allele frequency in all variants evaluated, except for *UCP3*, which was 12%. The sample was divided into two groups: cases with excess weight, with BMI p ≥85.0th (*n =* 267) and normal-weight controls with BMI p *<*85.0th (*n =* 738). A logistic regression analysis was done for each one of the 10 variants under the additive, dominant, and recessive models. The minor allele additive model showed that rs699 in AGT was significantly associated with an increase in the risk of excess weight, whereas rs1805097 in IRS2 was significantly associated with normal weight. The association remained significant after adjusting for age, gender, and pubertal maturation (see Table 2). The *AGT*-rs699 (additive model *p =* 0.034, OR = 1.25, 95% CI = 1.01–1.54), the *IRS2*-rs1805097 (additive model *p =* 0.022, OR = 0.77 95% CI = 0.62–0.96). An identical association was also observed for the dominant model (Table 2, part A). Focusing on the subset of the samples with information on ancestry, we observed that the ancestral composition is on average 66.3 ± 5.8 (%) European with a range in individuals of 41.0–80.0%; 19.5 ± 4.2 (%) Amerindian with a range of 10.0–35.0%; and 14.2 ± 4.9 (%) African with a range of 3.8–57.9%. Participants did not show any significant differences in ancestry according to nutritional status (normal weight vs. overweight) (Table 3). The odds ratios for the logistic regression analysis, adjusting ancestry as a covariate, were similar to those observed for the total sample, although the *p* values were weaker because of the smaller sample size (Table 2, part B). In addition, linear regression analyses of the adiposity measures with the 10 variants are shown in Table 4. The AGT-rs699 and IRS2-rs1805097 variants presented an association with BMI, FB%, and WC; UCP3-rs1800849 and FTO-rs17817449 variants presented an association with BMI and WC. After adjustment for age, gender, and pubertal maturation (for FB% and WC adjusted for BMI), the AGT-rs699 and FTO rs17817449 variants showed positive associations with BMI, and the IRS rs1805097 variant showed a negative association with BMI (*p =* 0.036, *p =* 0.031, and *p =* 0.043 respectively), while the UCP3 rs1800849 showed a negative association with WC (*p =* 0.001) (Table 4). We did a similar analysis on the subset of the sample with information on ancestry. Our results showed that after adjusting for ancestry, associations between IRS2-rs1805097 and FTO-rs17817449 on IMC were not confirmed (Additional file 1: Table S4). However, the association between AGT-rs699 on BMI and UCP3-rs1800849 on WC was still observed after adjusting for ancestry. However, this might be due to the effect of sample size, because IRS2-rs1805097 retained its association in logistic regression analysis after adjusting for ancestry.

**Table 2:** Association between 10 selected genetic variants and the risk of excess weight (p ≥85.0th) in young people aged 10 to 18 years

					Part A				Part B				
Region	Genetic variant	GENE	A2/A1	TEST	OR*	SE	95% CI	*p* value	OR^†^	SE	95% CI	*p* value	Risk allele
1q42.21	rs699	*AGT*	C/T	ADD	1.25	0.108	1.011, 1.545	**0.0394**	1.31	0.137	1.005, 1.720	**0.0458**	T
				DOM	1.36	0.156	1.005, 1.855	**0.0449**	1.46	0.183	1.021, 2.097	**0.0383**	
				REC	1.29	0.205	0.865, 1.929	0.2108	1.42	0.248	0.873, 2.313	0.1575	
2q37.3	rs3842570	*CAPN10*	IND/DEL	ADD	0.90	0.104	0.730, 1.099	0.2950	0.86	0.126	0.673, 1.108	0.2489	–
				DOM	0.86	0.156	0.632, 1.165	0.3324	0.76	0.190	0.530, 1.117	0.1680	
				REC	0.87	0.189	0.601, 1.260	0.4612	0.87	0.221	0.564, 1.345	0.5333	
8p21.3	rs285	*LPL*	C/T	ADD	1.11	0.102	0.910, 1.356	0.3009	1.19	0.124	0.934, 1.521	0.1572	–
				DOM	1.33	0.169	0.955, 1.854	0.0939	1.58	0.199	1.071, 2.341	0.0212	
				REC	0.10	0.171	0.712, 1.394	0.9822	1.021	0.205	0.682, 1.526	0.9213	
9q31.1	rs2230806	*ABCA1*	G/A	ADD	1.11	0.109	0.899, 1.379	0.3308	1.03	0.139	0.788, 1.362	0.7985	–
				DOM	1.31	0.154	0.968, 1.770	0.0756	1.25	0.181	0.882, 1.798	0.2037	
				REC	0.88	0.222	0.573, 1.369	0.5836	0.91	0.256	0.554, 1.515	0.7348	
10q25.2	rs7903146	*TCF7L2*	C/T	ADD	0.99	0.121	0.783, 1.260	0.9603	0.76	0.187	0.530, 1.106	0.1554	–
				DOM	1.04	0.149	0.775, 1.390	0.8100	0.87	0.175	0.618, 1.228	0.4318	
				REC	0.81	0.322	0.432, 1.523	0.5140	0.60	0.367	0.293, 1.242	0.1706	
11q13.4	rs1800849	*UCP3*	C/T	ADD	0.96	0.165	0.697, 1.332	0.8220	1.24	0.419	0.545, 2.826	0.6061	–
				DOM	0.95	0.178	0.672, 1.351	0.7911	1.10	0.210	0.729, 1.660	0.6494	
				REC	1.07	0.6915	0.276, 4.159	0.9196	1.51	0.837	0.293, 7.820	0.6202	
13q34	rs1805097	*IRS2*	G/A	ADD	0.77	0.1123	0.622, 0.966	**0.0222**	0.95	0.144	0.718, 1.265	0.7405	G
				DOM	0.67	0.1502	0.502, 0.905	**0.0090**	0.71	0.176	0.506, 1.012	**0.0582**	
				REC	0.85	0.2253	0.546, 1.321	0.4691	1.12	0.271	0.657, 1.909	0.6757	
16q12.2	rs17817449	*FTO*	T/G	ADD	1.17	0.1133	0.933, 1.455	0.1701	1.14	0.153	0.849, 1.548	0.3702	–
				DOM	1.17	0.1478	0.875, 1.561	0.2887	1.07	0.173	0.761, 1.503	0.6968	
				REC	1.36	0.2527	0.832, 2.239	0.2186	1.30	0.294	0.732, 2.322	0.3677	
16q13	rs708272	*CETP*	C/T	ADD	0.92	0.1107	0.741, 1.144	0.4594	0.94	0.135	0.727, 1.239	0.7021	–
				DOM	0.83	0.1514	0.619, 1.120	0.2306	0.82	0.178	0.584, 1.174	0.2899	
				REC	1.06	0.2168	0.692, 1.618	0.7945	1.01	0.252	0.617, 1.659	0.0622	
17q23.3	rs4340	*ACE*	IN/DEL	ADD	0.95	0.1029	0.781, 1.169	0.6571	0.96	0.120	0.762, 1.222	0.7669	–
				DOM	0.98	0.1686	0.706, 1.367	0.9270	1.00	0.197	0.681, 1.475	0.9902	
				REC	0.90	0.1708	0.643, 1.257	0.5349	0.90	0.199	0.612, 1.339	0.6208	

The ORs are based on the major allele homozygotes as reference. The significant *p* values (*p <* 0.05) are given in bold type

*A2* major allele, *A1* minor allele, *ADD* additive (major allele homozygotes vs. heterozygotes vs. minor allele homozygotes), *DOM* dominant (major allele homozygotes vs. heterozygotes + minor allele homozygotes), *REC* recessive (major allele homozygotes + heterozygotes vs. minor allele homozygotes), *OR* odds ratio, *CI* confidence interval

*Adjusted for age, gender, and pubertal maturation

^†^Adjusted for age, gender, pubertal maturation, and individual ancestry proportions

**Table 3:** Mean (standard deviation) of genetic ancestry percentages for nutritional status

	BMI p <85.0th *n =* 337	BMI p ≥85.0th *n =* 235	*p* value
European	66.2 (5.6)	66.7 (6.3)	0.327
Amerindian	19.6 (4.3)	19.3 (4.2)	0.439
African	14.4 (4.7)	14.1 (5.4)	0.552

Mean and standard deviation values are obtained from inferred individual genetic ancestry components (measured in percentage) for the subset of the samples. Student *t* test

**Table 4:** Association with anthropometric measures at 10 selected genetic variants in young people aged 10 to 18 years

			Log BMI (kg/m^2^)^a^				Log BF %^b^				Log waist circumference (cm)^b^			
SNP	MAF	TEST	ß coefficient	SE	95% CI	*p* value	ß coefficient	SE	95% CI	*p* value	ß coefficient	SE	95% CI	*p* value
rs699	T	ADD	0.007	0.003	0.000, 0.013	**0.036**	0.005	0.004	−0.003, 0.013	0.210	0.0008	0.000	−0.001, 0.002	0.365
rs3842570	D	ADD	−0.000	0.003	−0.006, 0.006	0.973	0.003	0.004	−0.005, 0.011	0.440	−0.0004	0.000	−0.002, 0.001	0.628
rs285	T	ADD	0.002	0.003	−0.003, 0.008	0.406	−0.002	0.004	−0.010, 0.005	0.577	−0.0000	0.000	−0.001, 0.001	0.994
rs2230806	A	ADD	0.003	0.003	−0.003, 0.010	0.286	−0.002	0.004	−0.010, 0.006	0.686	−0.0009	0.001	−0.002, 0.000	0.319
rs7903146	T	ADD	−0.001	0.003	−0.008, 0.006	0.851	0.002	0.005	−0.007, 0.011	0.653	−0.0003	0.001	−0.002, 0.001	0.772
rs1800849	T	ADD	−0.008	0.005	−0.017, 0.001	0.104	−0.005	0.006	−0.017, 0.006	0.373	−0.0045	0.001	−0.007, −0.001	**0.001**
rs1805097	A	ADD	−0.006	0.003	−0.013, −0.000	**0.043**	−0.005	0.004	−0.013, 0.003	0.200	0.0000	0.000	−0.001, 0.002	0.951
rs17817449	G	ADD	0.007	0.003	0.001, 0.014	**0.031**	−0.000	0.004	−0.009, 0.008	0.907	0.0011	0.001	−0.000, 0.003	0.287
rs708272	T	ADD	−0.013	0.003	−0.008, 0.005	0.672	−0.004	0.004	−0.012, 0.004	0.351	−0.0007	0.001	−0.002, 0.001	0.434
rs4340	D	ADD	−0.000	0.003	−0.006, 0.006	0.954	−0.001	0.004	−0.008, 0.007	0.877	−0.0009	0.000	−0.002, 0.000	0.316
GRS-3		ADD	0.020	0.007	0.006, 0.033	0.004	–	–	–	–	–	–	–	–

The significant *p* values (*p <* 0.05) are given in bold

*MAF* minor allele frequency calculated using the data from all the subjects in the analysis, *ADD* additive, *CI* confidence interval, *GRS* genetic risk score

^a^Adjusted for age, sex, and pubertal maturation

^b^Adjusted for age, sex, pubertal maturation, and BMI

**Fig. 1:**
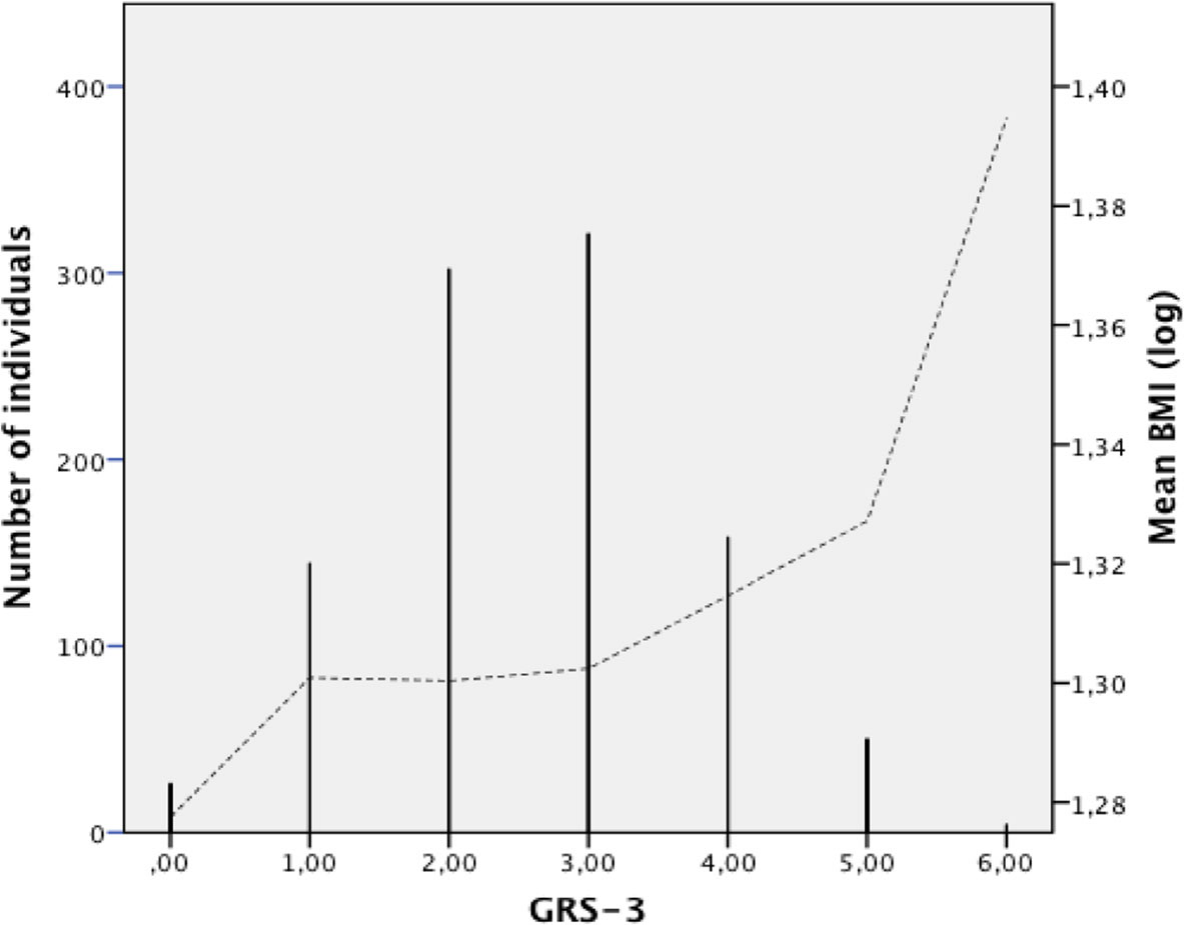
Distribution of the GRS-3 and cumulative effects of the alleles from the three BMI susceptibility variants on log-transformed BMI value

A GRS was generated for every individual by counting the number of alleles associated with excess weight (AGT-rs699 T and IRS2-rs1805097 G; range 0–4, (GRS-2)) and BMI measurement (AGT-rs699 T, IRS2-rs1805097 G and FTO-rs17817449 G; range 0–6, (GRS-3)). The association of the GRS-2 and the risk of excess weight showed that each additional effect allele was associated with a 1.26-fold increased odds of excess weight (95% CI 1.08–1.47). The GRS-3 was normally distributed. 16.9% of the individuals carried one or fewer risk alleles, and 5% carried ≥6 (Fig. 1). The GRS-3, which examines the cumulative effects of the three SNPs, was significantly associated with BMI (*p =* 0.004, effect size 0.034 log-transformed/allele 95% CI 0.006–0.033)

.

### Interaction between genetic variants and socioeconomic and perinatal variables

We did not find any statistically significant association among the rs3842570, rs285, rs2230806, rs7903146, rs708272, and rs4340 with BMI and adiposity measurements in this population. We explored the possible interaction of the socioeconomic status and perinatal history for the 10 variants evaluated and the indicators related to excess weight. This study found no evidence of interaction with these variables. An additional text file shows this in more detail (Additional file 1: Table S5).

## Discussion

The clustering of hypertension, dyslipidemia, and insulin resistance with obesity suggests the presence of common factors influenced by both environmental and genetic factors. Previous studies between body fat and common cardiovascular disease risk factors have indicated the presence of some genetic pleiotropism [32]. Additionally, epidemiological studies and clinical settings have established that outcomes often differ between ethnicity, and are modulated by social and environmental conditions. In this study we explore the association of 10 metabolic syndrome candidate genes with excess weight and adiposity and evaluate the effect that socioeconomic and perinatal factors could have on these associations in a group of young people.

Of the 10 variants evaluated, associations with excess weight, or its quantitative measurements, were found for four polymorphisms. A missense SNP in AGT (rs699; M235T) was associated with excess weight and may be a causal candidate variant; AGT II is an important regulator of blood pressure; carriers of the threonine variant have higher values in blood pressure [33], which leads to an increased risk associated with hypertension disorders. Skov and coworkers argued that regulating the renin-angiotensin-aldosterone system (RAAS) was involved in metabolic processes and could explain the origin and complications of some disorders such as MetS [34]. The RAAS components are involved in complex ways in the development of obesity by conditions of satiety, energy expenditure, and growth and differentiation of adipocytes [35]. AGT is also highly expressed in white adipose tissue, second to the liver in terms of mRNA levels; one study showed that overexpression of AGT in adipocytes increased weight in murine models [36]. Several observations suggested a role of AGT in adipose tissue development since this tissue and isolated adipocytes contain RAAS components, leading to AngII [37]. The expression and secretion of AGT increases with differentiation and is higher in adipocytes compared to preadipocytes [38].

We showed that the missense SNP rs1805097 in IRS2 was associated with protection for excess weight, an association not previously described. This SNP leads to a Gly1057Asp. Previous studies showed the role of IRS-2 in the differentiation of preadipocytes from adipocytes through the upregulation of the specific transcriptional factors of expression, such as PPARγ and C/EBP [39]. The variant is associated with diabetes in the adult population. However, studies from Italy and Pima Indians showed that this variant contributed to diabetes risk in obese individuals, but lowered risk for type 2 diabetes in normal-weight individuals, suggesting a possible interaction between gene and the environment [40, 41]. Kilpeläinen et al. [42] reported a locus near *IRS1* which was associated with a decrease in BF but with an impaired metabolic profile, including increased insulin resistance, dyslipidemia, risk of diabetes and coronary artery disease, and decreased adiponectin levels.

Different polymorphisms in the *FTO*-gene intron 1 have been associated with obesity or with BMI in genomic scans as well as in models of simple association in different populations [43, 44]. Recently, it was reported that the intron 1 polymorphisms function in regulatory elements of the expression of the *IRX3* gene. *IRX3* encodes a transcription factor highly expressed in the brain consistent with the role in regulating energy metabolism and feeding behavior [45]. As in other studies, we found an association with BMI in which the mean BMI was increased 0.33 kg/m^2^ per allele G [46].

UCP3 participates in thermogenesis; a decrease in the expression or function of this protein may reduce energy expenditure and increase its storage as fat. A promoter region variant −55 CT (rs1800849) is situated 6 bp from the TATA box and 4 bp from a DR1 site, which is a part of a retinoic acid response element [47]. The functions described for UCP3 and the results of variant association have been reported for children and adults in Caucasian and Korean populations [6, 48]. Liu et al. [6] found a statistically significant association between the carriers of the T allele and a lower BMI. Our study did not show an association with BMI, but it did show a statistically significant difference with a lower waist circumference.

With the exception of rs17817449, no association of these variants with BMI or waist circumference was found in the Genetic Investigation of ANthropometric Traits (GIANT) Consortium [49]. A steady genetic effect across population means that genetic variants reflect a common, final biological effect on individuals. However, associations may be altered by age, environmental exposures, interaction with other genes, effects of the differences in the allele frequencies, and the linkage disequilibrium patterns in admixed populations.

Evidence shows an interaction among the variants of obesity with environmental factors, as has been found for *FTO*, in which the lack of physical activity and less education potentiate the effect of its variants in the increase of obesity [18, 23]. Similarly, a variant of the *APOA2* gene increases the association with obesity in those consuming high levels of saturated fatty acids [50]. Another study reported the influence of maternal education on the effect of a variant of the neuromedin B gene on obesity [19]. Based on these studies, we evaluated environments that conducive to possible interaction, such as social status, education, history of breastfeeding, and birth weight. However, no associations between the 10 variants and these factors were found, possibly due to sample size.

However, significant association was found between the education of parents with excess weight in this study similar to that observed in Bangladesh and India, which reported that higher levels of education and greater income were associated with the increase in the rates of obesity [16, 17]. Although not monitored here, families with higher levels of education and better economic incomes may consume higher levels of fast foods and, coupled with the availability of video games, creates sedentary environments that favor the development of obesity.

Birth weight and length are considered to be high-risk variables for excess weight [51]. The study by Loaiza et al. [52] in Chilean children, reported a direct relationship between high birth weight and the risk of obesity in school age. Evidence exists on the effect of the fetal environment and the epigenetic remodeling in fetal genes that regulate or participate in the energy metabolism that will be expressed in obesogenic environments [53]. In this study, birth weight showed a borderline significance (*p =* 0.056).

Family history of obesity meets both genetic risk factors as well as environmental or cultural factors. Heritability estimates for obesity range between 50 and 80% [54] based on the concordance in monozygotic compared to dizygotic twins.

Several limitations of this study must be considered: the sample size may be relatively small for a predictive clinical-genomic model. In addition, genotype-environment interactions would require a larger population to confirm these results. Furthermore, this study only considered the association of a single variant in each gene with BMI, a widely used but imprecise indicator of adiposity. X-ray dual absorptiometry produces more precise data on adiposity but because of cost its use is limited. A wide variety of other environmental factors and social determinants were not explored in this study which could affect the associations.

Despite these limitations, this study contributes to the literature on understanding childhood obesity, showing associations between the family socioeconomic level and anthropometric, health, and genetic measurements. Although the study reported here, like most association studies, focused on single SNP associations, excess weight is a complex trait produced by combinations of many gene-gene and gene-environment interactions. The pathway for the translation and integration of genomics from the laboratory into everyday medicine remain largely unfulfilled, and the clinical utility has been debated recently [55, 56]. Genetic studies should be considered as a first step in the understanding of the molecular basis of traits or complex diseases but requires a more comprehensive view integrating phenotypic and genotypic factors with environmental and metabolic factors. We believe that the results provide further insights regarding the potential modulating effect of certain genotypes to weight reduction treatments.

## Conclusions

We found that the SNPs of *AGT* rs699 and *IRS2* rs1805097 showed significant association with overweight (BMI p >85). The variants rs699, rs1805097, and rs17817449 were significantly associated with BMI and the variant of *UCP3* rs1800849, with waist circumference. In addition, we found that the level of parent education, family histories of obesity, hypertension, dyslipidemia, and a minor duration of breastfeeding contribute to excess weight; however, no effect of these socioeconomic and perinatal factors was found on genetic associations.

Although obesity is a complex disease, the implementation of personalized treatments using genotypes could be tested in this condition if our findings are confirmed.

Future research should explore, in a larger sample size, the interaction of other environmental factors such as lifestyles and diet habits, in addition to a larger number of polymorphisms in each gene. This might help explain the relationship between the genotype and phenotype, which may aid clinicians for developing treatments or predicting outcomes.

## Additional file

Additional file 1: Table S1. Sequence of primer used to PCR. **Table S2.** Ancestral informative Markers (AIMs), allelic frequencies and delta (δ) of frequencies in the three ancestral populations (European, African and Amerindian). **Table S3.** Characteristics of the study population, stratified according to gender. **Table S4.** Association with anthropometric measures at 10 selected genetic variants on the subset of the samples with available ancestry information. **Table S5.** Interaction between SNP and socioeconomic and perinatal factors in determining body mass index. **Figure S1.** The histogram of body mass index, body fat percentage and waist circumference by gender. (DOCX 123 kb)

## Abbreviations

%BF: Body fat percentage
MetS: Metabolic syndrome
ABCA1: ATP-binding cassette, sub-family A (ABC1), member 1
ACE: Angiotensin-converting enzyme
AGT: Angiotensinogen
AIMs: Ancestry informative markers
ANOVA: Analysis of variance
APO2: Apolipoprotein A-II
BMI: Body mass index
CAPN10: Calpain 10
CETP: Cholesteryl ester transfer protein
CIs: Confidence intervals
ENSIN: National Nutritional Situation Survey, 2005
FTO: Fat mass and obesity-associated
GIANT Consortium: Genetic Investigation of ANthropometric Traits Consortium
GRS: Genetic risk score
IRS2: Insulin receptor substrate 2
LPL: Lipoprotein lipase
OR: Odds ratio
SNP: Single nucleotide polymorphism
TCF7L2: Transcription factor 7-like 2
UCP3: Uncoupling Protein-3
WC: Waist circumference
WHO: World Health Organization
