# Supplementary material for: Examining for an association between candidate gene polymorphisms in the metabolic syndrome components on excess weight and adiposity measures in youth: a cross-sectional study
Source: Genes Nutr. 2017 Jul 4;12:19. doi: 10.1186/s12263-017-0567-1 (PMC5496328; doi:10.1186/s12263-017-0567-1)

Additional file

**Table S1.** Sequence of primer used to PCR

| Gene | Primer sequence: | Enzyme |
| --- | --- | --- |
| AGT-rs699 | F5’CCGTTTGTGCAGGGCCTGGCTCTCT 3’  R5'CAGGGTGCTGTCCACACTGGACCCC 3’ | Tth111 |
| CAPN10-rs3842570 | F5´ GTTTGGTTCTCTTCAGCGTGGAG3´  R5´CATGAACCCTGGCAGGGTCTAAG 3´ | In/Del |
| LPL-rs285 | F5´AGGCTTCACTCATCCGTGCCTCC 3´  R5´TTATGCTGCTTTAGACTCTTGTC 3´ | *PvuII* |
| ABCA1-rs2230806 | F5´GTATTTTTGCAAGGCTACCAGTTACAT 3´  R5´GATTGGCTTCAGGATGTCCATGTTGG 3´ | *XagI* |
| TCF7L2-rs7903146 | F5´TTAGAGAGCTAAGCACTTTTTAGGTA3´  R5´ACTAAGTTACTTGCCTTCCCTG3´ | *RsaI* |
| UCP3-rs1800849 | F5´ GAGACTATATTAAAGCACCCCGGGTCAAGAGGAC 3´  R5´ TCTGCTGCTTCTGGCTTGGCACTGGTCTTATACACCC 3´ | *SmaI* |
| IRS2-rs1805097 | F5’ GCTCCCCCAAGTCTCCTAA 3’  R5’ CTCAGCCTCTTCACGCCC 3’ | *HaeII* |
| FTO-rs17817449 | F5´AGGACCTCCTATTTGGGACA3´  R5´AGCTTCCATGGCTAGCATTA3 | *AlwN I* |
| CETP-rs708272 | F5´CACTAGCCCAGAGAGAGGAGTGCC 3´  R5´ CTGAGCCCAGCCGCACACTAA C 3´ | *TaqI* |
| ACE-rs4340 | F5´CTGGAGACCACTCCCATCCTTTCT 3´  R5´GATGTGGCCATCACATTCGTCAGAT 3´ | IN/DEL |

**Table S2.** Ancestral informative Markers (AIMs), allelic frequencies and delta (δ) of frequencies in the three ancestral populations (European, African and Amerindian)

| AIM | Chr | Phys. Loc.  (bp) | Freq  Eu | Freq  Am | Freq  Af | δ  (Af-Eu) | δ  (Af-Am) | δ  (Eu-Am) |
| --- | --- | --- | --- | --- | --- | --- | --- | --- |
| *MID1752* | 1 | 55048581 | 0.290 | 0.920 | 0.560 | 0.270 | 0.360 | **0.630** |
| *FYNULL* | 1 | 164822103 | 0.998 | 1.000 | 0.001 | **0.997** | **0.999** | 0.002 |
| *AT3* | 1 | 170618399 | 0.282 | 0.061 | 0.858 | **0.576** | **0.797** | 0.221 |
| *MID1386* | 1 | 244094748 | 0.730 | 0.070 | 0.770 | 0.040 | **0.700** | **0.660** |
| *NBC4* | 2 | 74296877 | 0.683 | 0.947 | 0.513 | 0.170 | **0.430** | 0.264 |
| *MID921* | 3 | 708993 | 0.090 | 0.060 | 0.690 | **0.600** | **0.630** | 0.030 |
| *Ya5ACA1100* | 3 | 156131069 | 1.000 | 1.000 | 0.450 | **0.550** | **0.550** | 0.000 |
| *D1* | 3 | 179593741 | 0.011 | 0.500 | 0.256 | 0.245 | 0.244 | **0.489** |
| *Ya5ACA1184* | 4 | 41782815 | 0.688 | 0.947 | 0.158 | **0.530** | **0.789** | 0.259 |
| *GC-1F* | 4 | 72983369 | 0.156 | 0.339 | 0.853 | **0.697** | **0.514** | 0.183 |
| *MID1586* | 4 | 88316243 | 0.420 | 0.260 | 0.960 | **0.540** | **0.700** | 0.160 |
| *MID52* | 4 | 201758344 | 0.840 | 0.140 | 0.740 | 0.100 | **0.600** | **0.700** |
| *Ya5ACA1153* | 4 | 181786436 | 0.269 | 0.833 | 0.306 | 0.037 | **0.527** | **0.564** |
| *MID817* | 5 | 11400042 | 0.650 | 0.130 | 0.960 | 0.310 | **0.830** | **0.520** |
| *MID1039* | 5 | 34009694 | 0.270 | 0.830 | 0.980 | **0.710** | 0.150 | **0.560** |
| *MID1358* | 5 | 35655730 | 0.060 | 0.040 | 0.800 | **0.740** | **0.760** | 0.020 |
| *MID856* | 5 | 65320596 | 0.150 | 0.690 | 0.660 | **0.510** | 0.030 | **0.540** |
| *MID944* | 5 | 92340215 | 0.390 | 0.950 | 0.890 | **0.500** | 0.060 | **0.560** |
| *pAlu6-17534722* | 6 | 17580264 | 0.750 | 0.375 | 0.125 | **0.625** | 0.250 | 0.375 |
| *MID108* | 6 | 32808464 | 0.320 | 0.040 | 0.580 | 0.260 | **0.540** | 0.280 |
| *MID104* | 6 | 32810633 | 0.350 | 0.110 | 0.560 | 0,210 | **0.450** | 0.240 |
| *MID2062* | 6 | 40194876 | 0.290 | 0.930 | 0.410 | 0.120 | **0.520** | **0.640** |
| *Ya5ACA1702* | 7 | 42614187 | 0.800 | 1.000 | 0.500 | 0.300 | **0.500** | 0.200 |
| *Ya5ACA1611* | 7 | 113740756 | 0.611 | 1.000 | 0.211 | 0.400 | **0.789** | **0.389** |
| *LPL* | 8 | 19859469 | 0.492 | 0.442 | 0.971 | **0.479** | **0.529** | 0.050 |
| *Ya5aca1861* | 9 | 26502940 | 0.417 | 0.889 | 0.875 | **0.458** | 0.014 | **0.472** |
| *MID1780* | 11 | 35103909 | 0.230 | 0.690 | 0.740 | **0.510** | 0.050 | **0.460** |
| *B65* | 11 | 89572171 | 0.489 | 0.270 | 0.830 | 0.341 | **0.560** | 0.219 |
| *DRD2* | 11 | 112796798 | 0.670 | 0.045 | 0.135 | **0.535** | 0.090 | **0.625** |
| *APOA* | 11 | 123600000 | 0.925 | 0.977 | 0.420 | **0.505** | **0.557** | 0.052 |
| *MID1723* | 12 | 79547087 | 0.180 | 0.150 | 0.900 | **0.720** | **0.750** | 0.030 |
| *RB2300* | 13 | 47776293 | 0.315 | 0.175 | 0.926 | **0.611** | **0.751** | 0.140 |
| *MID2264* | 13 | 62676757 | 0.300 | 1.000 | 1.000 | **0.700** | 0.000 | **0.700** |
| *MID2269* | 13 | 85138935 | 0.400 | 0.900 | 0.900 | **0.500** | 0.000 | **0.500** |
| *OCA2* | 15 | 25909368 | 0.746 | 0.488 | 0.115 | **0.631** | 0.373 | 0.258 |
| *MID818* | 16 | 2140623 | 0.780 | 0.980 | 0.090 | **0.690** | **0.890** | 0.200 |
| *PV92* | 16 | 87700000 | 0.152 | 0.792 | 0.225 | 0.073 | **0.567** | **0.640** |
| *Ya5_435* | 17 | 64599263 | 0.400 | 1.000 | 0.200 | 0.200 | **0.800** | **0.800** |
| *SB19.3* | 19 | 27200000 | 0.903 | 0.645 | 0.415 | **0.488** | 0.230 | 0.258 |
| *MID154* | 20 | 32140667 | 0.250 | 0.140 | 0.820 | **0.570** | **0.680** | 0.110 |

Chr: Chromosome; Phys. Loc: physical location; Freq: Allele frequency; Eu: European; Am: Amerindian; Af: African; δ: Differences among the continental populations. Equals to differences between pairs of continental populations. δ >0.40 is shown in bold.

**Table S3**. Characteristics of the study population, stratified according to gender

| Variable | All | Men | Women | p-value |
| --- | --- | --- | --- | --- |
| Socioeconomic Status ^a^  Low  Medium  High | 42.3  37.5  20.3 | 43.6  37.6  18.8 | 41.1  37.3  21.5 | 0.505 |
| Maternal education, year ^a^  0 -6  + 6 – 12  +12 | 13.4  48.0  36.8 | 13.1  50.7  36.2 | 13.6  45.6  40.8 | 0.232 |
| Paternal education, year ^a^  0 – 6  +6 – 12  +12 | 13.7  46.4  39.9 | 13.0  48.8  38.2 | 14.3  44.3  41.4 | 0.373 |
| Pubertal maturation ^a^  Prepubertal  Pubertal  Postpubertal | 18.2  28.4  53.4 | 22.0  26.8  51.2 | 15.0  29.8  55.2 | **0.011** |
| Family history ^a^  Obesity  Type-2 diabetes  Gestational diabetes  Hypertension  Dyslipidemia | 40.9  57.3  2.7  77.8  62.1 | 42.0  59.6  2.0  78.6  62.2 | 40.0  55.4  3.3  77.2  61.9 | 0.498  0.161  0.195  0.571  0.929 |
| Birth weight, (g) ^a^  < 2500  ≥2500 – 4000  >4000  Maternal breastfeeding ^a^ | 8.5  85.8  5.7  92.9 | 7.2  86.3  6.5  92.8 | 9.6  85.4  5.1  93.1 | 0.272  0.851 |
| Duration of breastfeeding, (months) ^a^  0 -1  >1-3  >3 – 6  > 6 | 7.1  21.8  21.4  49.7 | 7.3  21.0  20.6  51.2 | 7.0  22.5  22.1  48.4 | 0.795 |
| BMI ^a^  Normal–weight (p < 85.0th)  Overweight (p ≥ 85.0th) | 74.8  25.2 | 73.2  26.8 | 76.2  23.8 | 0.250 |
| Anthropometry ^b^  Weight, kg  BMI, kg/m2  BF%  Waist circumference, (cm) | 50.3 (13.3)  40.4 (3.5)  24.8 (8.9)  67.7 (8.4) | 52.6 (15.6)  20.3 (3.8)  19.0 (8.7)  69.7 (9.9) | 48.3 (10.6)  20.4 (3.32)  29.7 (5.5)  66.1 (7.5) | **0.000**  0.193  **0.000**  **0.000** |

Data is shown as percentage (%) or average ± standard deviation. ^a^ Pearson´s Chi-square, ^b^ T-Student test. The significant p-values (p < 0.05) are given in bold. BMI: Body Mass Index, BF%: Body fat percentage

**Table S4**. Association with anthropometric measures at 10 selected genetic variants on the subset of the samples with available ancestry information

|  |  | BMI (kg/m^2^)^a^ | | | |  | BF %^b^ | | | |  | Waist circumference (cm)^b^ | | | |
| --- | --- | --- | --- | --- | --- | --- | --- | --- | --- | --- | --- | --- | --- | --- | --- |
| SNP | MAF | ß-coefficient | SE | 95% CI | P-value |  | ß-coefficient | SE | 95% CI | P-value |  | ß-coefficient | SE | 95% CI | P-value |
| rs699 | T | 0.009 | 0.004 | 0.002, 0.017 | **0.0186** |  | 0.005 | 0.005 | -0.005, 0.0152 | 0.338 |  | 0.001 | 0.001 | -0.001, 0.003 | 0.469 |
| rs3842570 | D | -0.003 | 0.004 | -0.010, 0.004 | 0.4272 |  | 0.002 | 0.005 | -0.008, 0.012 | 0.661 |  | 0.001 | 0.001 | -0.001, 0.003 | 0.503 |
| rs285 | T | 0.004 | 0.004 | -0.003, 0.011 | 0.287 |  | -0.003 | 0.005 | -0.0122, 0.007 | 0.576 |  | 0.000 | 0.001 | -0.002, 0.003 | 0.667 |
| rs2230806 | A | 0.005 | 0.004 | -0.003, 0.013 | 0.196 |  | 0.002 | 0.005 | -0.008, 0.012 | 0.696 |  | -0.002 | 0.001 | -0.004, 0.001 | 0.171 |
| rs7903146 | T | -0.008 | 0.004 | -0.016, 0.000 | 0.060 |  | 0.006 | 0.006 | -0.005, 0.017 | 0.290 |  | 0.001 | 0.001 | -0.002, 0.003 | 0.680 |
| rs1800849 | T | -0.001 | 0.006 | -0.013, 0.010 | 0.835 |  | -0.010 | 0.008 | -0.025, 0.006 | 0.216 |  | -0.005 | 0.002 | -0.008, -0.001 | **0.011** |
| rs1805097 | A | -0.005 | 0.004 | -0.013, 0.003 | 0.189 |  | -0.001 | 0.005 | -0.011, 0.009 | 0.859 |  | -0.000 | 0.001 | -0.003, 0.002 | 0.832 |
| rs17817449 | G | 0.004 | 0.004 | -0.004, 0.012 | 0.302 |  | 0.005 | 0.005 | -0.006, 0.015 | 0.368 |  | 0.001 | 0.001 | -0.002, 0.003 | 0.568 |
| rs708272 | T | -0.001 | 0.004 | -0.009, 0,006 | 0.725 |  | -0.004 | 0.005 | -0.014, 0.006 | 0.472 |  | -0.000 | 0.001 | -0.003, 0.002 | 0.751 |
| rs4340 | D | 0.001 | 0.004 | -0.006, 0.008 | 0.837 |  | 0.005 | 0.005 | -0.004, 0.015 | 0.258 |  | -0.001 | 0.001 | -0.003, 0.002 | 0.575 |

MAF: minor allele frequency calculated using the data from all the subjects in the analysis; ADD, Additive; CI, confidence interval. ^a^Adjusted for age, sex, and puberal maduration. ^b^Adjusted for age, sex, puberal maduration and BMI. The significant p-values (p < 0.05) are given in bold type

**Table S5**. Interaction between SNP and socio-economic and perinatal factors in determining Body Mass Index

|  | Social stratum | | | | | | | | | Maternal education year | | | | | | |
| --- | --- | --- | --- | --- | --- | --- | --- | --- | --- | --- | --- | --- | --- | --- | --- | --- |
|  | Low | | Medium | | | high | | |  | 0-6 years | | >6-12 years | | >12 years | |  |
|  | OR | 95%IC | OR | 95%IC | | OR | 95%IC | | P for interation | OR | 95%IC | OR | 95%IC | OR | 95%IC | P for interation |
| AGT |  |  |  |  | |  |  | |  |  |  |  |  |  |  |  |
| C/C | 1.00 | reference | 1.21 | 0.71-2.08 | | 2.02 | 0.97-4.17 | | 0.735 | 1.00 | reference | 1.54 | 0.66-3.59 | 1.30 | 0.53-3.16 | 0.554 |
| C/T | 1.64 | 1.00-2.69 | 1.39 | 0.83-2.35 | | 1.90 | 1.06-3.43 | |  | 0.88 | 0.32-2.42 | 1.99 | 0.86-4.59 | 2.31 | 0.99-5.36 |  |
| T/T | 1.37 | 0.61-3.08 | 1.65 | 0.80-3.41 | | 2.50 | 1.21-5.18 | |  | 0.63 | 0.07-5.96 | 1.76 | 0.67-4.60 | 2.79 | 1.09-7.10 |  |
| CAPN10 |  |  |  |  | |  |  | |  |  |  |  |  |  |  |  |
| I/I | 1.00 | reference | 0.82 | 0.46-1.47 | | 1.77 | 0.94-3.34 | | 0.162 | 1.00 | reference | 1.74 | 0.70-4.33 | 1.83 | 0.73-4.61 | 0.853 |
| I/D | 0.81 | 0.48-1.38 | 1.03 | 0.62-1.73 | | 0.94 | 0.50-1.75 | |  | 0.58 | 0.18-1.87 | 1.50 | 0.62-3.66 | 1.59 | 0.65-3.92 |  |
| D/D | 0.81 | 0.44-1.50 | 0.61 | 0.29-1.27 | | 1.96 | 0.81-4.73 | |  | 0.87 | 0.26-2.90 | 1.23 | 0.47-3.21 | 1.83 | 0.67-5.04 |  |
| LPL |  |  |  |  | |  |  | |  |  |  |  |  |  |  |  |
| C/C | 1.00 | reference | 0.89 | 0.46-1.72 | | 1.35 | 0.64-2.86 | | 0.901 | 1.00 | reference | 2.36 | 0.76-7.34 | 2.53 | 0.81-7.89 | 0.975 |
| C/T | 1.31 | 0.74-2.32 | 1.26 | 0.70-2.26 | | 1.98 | 1.04-3.75 | |  | 1.91 | 0.55-6.63 | 3.10 | 1.04-9.28 | 3.67 | 1.22-11.04 |  |
| T/T | 0.97 | 0.50-1.87 | 1.32 | 0.68-2.54 | | 1.92 | 0.83-4.45 | |  | 1.19 | 0.28-5.03 | 2.82 | 0.92-8.70 | 3.22 | 1.00-10.38 |  |
| ABCA1 |  |  |  |  | |  |  | |  |  |  |  |  |  |  |  |
| G/G | 1.00 | reference | 0.79 | 0.45-1.40 | | 1.59 | 0.86-2.94 | | 0.523 | 1.00 | reference | 1.50 | 0.63-3.60 | 1.89 | 0.79-4.54 | 0.902 |
| A/G | 1.23 | 0.74-2.04 | 1.56 | 0.94-2.57 | | 1.80 | 0.98-3.29 | |  | 1.20 | 0.42-3.41 | 2.39 | 1.02-5.60 | 2.62 | 1.10-6.21 |  |
| A/A | 1.12 | 0.56-2.23 | 0.83 | 0.35-1.94 | | 2.23 | 0.78-6.41 | |  | 0.58 | 0.11-3.09 | 2.06 | 0.78-5.46 | 2.22 | 0.77-6.43 |  |
| TCF7L2 |  |  |  |  | |  |  | |  |  |  |  |  |  |  |  |
| C/C | 1.00 | reference | 0.83 | 0.53-1.30 | | 1.69 | 0.99-2.78 | | 0.053 | 1.00 | reference | 1.75 | 0.91-3.38 | 2.19 | 1.12-4.30 | 0.576 |
| C/T | 0.81 | 0.49-1.34 | 1.32 | 0.83-2.09 | | 1.46 | 0.81-2.65 |  | | 0.69 | 0.23-2.12 | 2.13 | 1.07-4.24 | 2.06 | 1.02-4.14 |  |
| T/T | 1.81 | 0.71-4.62 | 0.58 | 0.20-1.67 | | 0.33 | 0.04-2.60 | |  | 2.65 | 0.38-18.57 | 1.36 | 0.48-3.82 | 1.51 | 0.46-4.95 |  |
| UCP3 |  |  |  |  | |  |  | |  |  |  |  |  |  |  |  |
| C/C | 1.00 | reference | 0.94 | 0.65-1.37 | | 1.50 | 0.96-2.33 | | 0.482 | 1.00 | reference | 1.99 | 1.08-3.68 | 2.33 | 1.25-4.35 | 0.500 |
| C/T | 0.73 | 0.39-1.34 | 1.09 | 0.63-1.89 | | 1.47 | 0.75-2.88 | |  | 0.96 | 0.31-2.97 | 2.19 | 1.06-4.53 | 2.19 | 0.99-4.50 |  |
| T/T | 1.65 | 0.37-7.34 | 0.00 | | | 0.00 | | |  | --- | | 1.15 | 0.13-10.48 | 1.95 | 0.16-23.95 |  |
| IRS2 |  |  |  |  | |  |  | |  |  |  |  |  |  |  |  |
| G/G | 1.00 | reference | 0.89 | 0.53-1.49 | | 1.25 | 0.70-2.24 | | 0.554 | 1.00 | reference | 2.81 | 1.22-6.50 | 2.28 | 0.96-5.41 | 0.173 |
| G/A | 0.53 | 0.32-0.87 | 0.66 | 0.41-1.08 | | 1.15 | 0.65-2.05 | |  | 0.78 | 0.27-2.26 | 1.31 | 0.56-3.05 | 2.17 | 0.94-5.05 |  |
| A/A | 0.79 | 0.39-1.62 | 0.64 | 0.30-1.35 | | 0.68 | 0.23-1.96 | |  | 1.81 | 0.44-7.40 | 1.66 | 0.63-4.40 | 1.62 | 0.57-4.57 |  |
| FTO |  |  |  |  | |  |  | |  |  |  |  |  |  |  |  |
| T/T | 1.00 | reference | 1.22 | 0.77-1.95 | | 1.45 | 0.83-2.53 | | 0.346 | 1.00 | reference | 2.00 | 0.97-4.12 | 2.26 | 1.08-4.71 | 0.397 |
| G/T | 1.16 | 0.71-1.88 | 1.17 | 0.71-1.94 | | 2.05 | 1.14-3.70 | |  | 1.47 | 0.56-3.91 | 2.08 | 1.00-4.35 | 2.53 | 1.19-5.37 |  |
| G/G | 2.14 | 0.99-4.63 | 0.82 | 0.32-2.09 | | 2.89 | 0.00-8.44 | |  | 0.00 | | 3.26 | 1.31-8.15 | 2.96 | 1.08-8.16 |  |
| CETP |  |  |  |  | |  |  | |  |  |  |  |  |  |  |  |
| C/C | 1.00 | reference | 0.90 | 0.53-1.54 | | 1.21 | 0.66-2.22 | | 0.79 | 1.00 | reference | 2.31 | 0.94-5.69 | 1.79 | 0.72-4.44 | 0.143 |
| C/T | 0.72 | 0.44-1.19 | 0.76 | 0.45-1.28 | | 1.17 | 0.64-2.13 | |  | 1.01 | 0.35-2.91 | 1.31 | 0.54-3.21 | 2.12 | 0.86-5.23 |  |
| T/T | 0.75 | 0.37-1.52 | 0.84 | 0.39-1.80 | | 2.05 | 0.74-5.69 | |  | 0.42 | 0.07-2.34 | 2.21 | 0.81-6.02 | 2.13 | 0.74-6.13 |  |
| ACE |  |  |  |  | |  |  | |  |  |  |  |  |  |  |  |
| I/I | 1.00 | reference | 0.94 | 0.50-1.78 | | 1.25 | 0.57-2.75 | | 0.73 | 1.00 | reference | 2.05 | 0.71-5.90 | 2.25 | 0.76-6.61 | 0.604 |
| I/D | 0.89 | 0.51-1.55 | 1.06 | 0.60-1.84 | | 1.39 | 0.74-2.60 | |  | 1.22 | 0.37-4.02 | 1.83 | 0.66-5.10 | 2.56 | 0.91-7.15 |  |
| D/D | 0.90 | 0.48-1.69 | 0.70 | 0.36-1.39 | | 1.66 | 0.79-349 | |  | 0.77 | 0.20-3.06 | 2.29 | 0.79-6.61 | 1.78 | 0.60-5.27 |  |
|  | Birth weight, (g) | | | | | | | | | Maternal breastfeeding | | | | | |  |
|  |  | < 2500 | ≥2500 – 4000 | | >4000 | | | |  | Yes | | Not | |  |  |  |
|  | OR | 95%IC | OR | 95%IC | | OR | 95%IC | | P for  interaction |  |  |  |  | P for Interaction | | |
| AGT |  |  |  |  | |  |  | |  |  |  |  |  | 0.393 | | |
| C/C | 1.00 | reference | 1.67 | 0.60-4.65 | | 4.73 | 1.11-20.20 | | 0.602 | 1.00 | reference | 1.08 | 0.40-2.91 |  | | |
| C/T | 2.15 | 0.59-7.86 | 2.34 | 0.85-6.44 | | 2.49 | 0.66-9.32 | |  | 1.28 | 0.91-1.79 | 2.59 | 1.20-5.63 |  | | |
| T/T | 1.16 | 0.19-7.23 | 2.66 | 0.92-7.71 | | 4.12 | 0.76-22.46 | |  | 1.40 | 0.88-2.25 | 4.38 | 1.24-15.48 |  | | |
| CAPN10 |  |  |  |  | |  |  | |  |  |  |  |  |  | | |
| I/I | 1.00 | reference | 0.93 | 0.33-2.62 | | 1.36 | 0.31-5.90 | | 0.145 | 1.00 | reference | 1.64 | 0.62-4.37 |  | | |
| I/D | 0.47 | 0.13-1.73 | 0.81 | 0.29-2.24 | | 2.35 | 0.64-8.60 | |  | 0.85 | 0.60-1.20 | 1.71 | 0.79-3.72 | 0..916 | | |
| D/D | 0.37 | 0.06-2.33 | 0.70 | 0.27-2.28 | | 0.22 | 0.02-2.16 | |  | 0.81 | 0.52-1.25 | 1.25 | 0.40-3.96 |  | | |
| LPL |  |  |  |  | |  |  | |  |  |  |  |  |  |  |  |
| C/C | 1.00 | reference | 2.22 | 0.49-10.11 | | 3.70 | 0.54-25.46 | | 0.867 | 1.00 | reference | 1.22 | 0.38-3.95 | 0.702 | | |
| C/T | 2.94 | 0.56-15.55 | 3.24 | 0.72-14.50 | | 4.66 | 0.84-25.95 | |  | 1.33 | 0.92-1.92 | 2.84 | 1.34-6.02 |  | | |
| T/T | 1.27 | 0.18-8.93 | 2.83 | 0.62-12.92 | | 4.86 | 0.76-30.97 | |  | 1.17 | 0.77-1.80 | 1.83 | 0.57-5.94 |  | | |
| ABCA1 |  |  |  |  | |  |  | |  |  |  |  |  |  |  |  |
| G/G | 1.00 | reference | 1.50 | 0.59-3.83 | | 3.91 | 1.11-13.76 | | 0.275 | 1.00 | reference | 2.04 | 0.86-4.87 | 0.571 | | |
| A/G | 1.31 | 0.38-4.58 | 2.20 | 0.87-5.57 | | 3.78 | 1.00-14.25 | |  | 1.43 | 1.02-2.00 | 2.63 | 1.25-5.51 |  | | |
| A/A | 2.99 | 0.43-20.55 | 1.75 | 0.64-4.79 | | 0.60 | 0.06-5.83 | |  | 1.20 | 0.74-1.96 | 0.74 | 0.08-6.88 |  | | |
| TCF7L2 |  |  |  |  | |  |  | |  |  |  |  |  |  |  |  |
| C/C | 1.00 | reference | 1.11 | 0.53-2.31 | | 2.50 | 0.88-7.09 | | 0.389 | 1.00 | reference | 1.63 | 0.82-3.22 | 0.571 | | |
| C/T | 0.29 | 0.06-1.45 | 1.27 | 0.60-2.67 | | 1.83 | 0.53-6.29 | |  | 1.07 | 0.78-1.48 | 2.38 | 0.99-5.74 |  | | |
| T/T | 2.13 | 0.27-16.64 | 0.98 | 0.36-2.66 | | 0.00 | | |  | 0.92 | 0.48-1.76 | 0.00 | |  | | |
| UCP3 |  |  |  |  | |  |  | |  |  |  |  |  |  |  |  |
| C/C | 1.00 | reference | 1.26 | 0.65-2.45 | | 1.87 | 0.72-4.81 | | 0.191 | 1.00 | reference | 1.44 | 0.78-2.66 | 0.218 | | |
| C/T | 0.24 | 0.03-2.04 | 1.30 | 0.63-2.70 | | 2.42 | 0.64-9.06 | |  | 0.92 | 0.63-1.33 | 3.20 | 1.01-10.17 |  | | |
| T/T | --- | | 0.96 | 0.18-5.25 | | --- | | |  | 0.76 | 0.15-3.70 | --- | |  | | |
| IRS2 |  |  |  |  | |  |  | |  |  |  |  |  |  |  |  |
| G/G | 1.00 | reference | 3.21 | 1.06-9.72 | | 6.22 | 1.59-24.27 | | 0.064 | 1.00 | reference | 2.87 | 1.23-6.67 | 0.355 | | |
| G/A | 2.65 | 0.72-9.69 | 2.10 | 0.70-6.35 | | 1.54 | 0.29-8.14 | |  | 0.70 | 0.50-0.97 | 0.95 | 0.41-2.18 |  | | |
| A/A | 0.00 |  | 2.15 | 0.66-6.95 | | 4.80 | 0.80-28.78 | |  | 0.76 | 0.46-1.24 | 0.78 | 0.16-3.76 |  | | |
| FTO |  |  |  |  | |  |  | |  |  |  |  |  |  |  |  |
| T/T | 1.00 | reference | 1.66 | 0.70-3.95 | | 1.75 | 0.51-6.05 | | 0.443 | 1.00 | reference | 1.28 | 0.55-3.00 | 0.454 | | |
| G/T | 0.95 | 0.26-3.51 | 1.71 | 0.71-4.12 | | 4.31 | 1.30-14.33 | |  | 1.04 | 0.75-1.44 | 2.62 | 1.23-5.59 |  | | |
| G/G | 4.37 | 0.76-25.2 | 2.17 | 0.81-5.79 | | 2.29 | 0.18-29.41 | |  | 1.41 | 0.83-2.42 | 1.68 | 0.33-8.62 |  | | |
| CETP |  |  |  |  | |  |  | |  |  |  |  |  |  |  |  |
| C/C | 1.00 | reference | 1.46 | 0.55-3.85 | | 2.79 | 0.77-10.08 | | 0.814 | 1.00 | reference | 2.34 | 1.04-5.25 | 0.392 | | |
| C/T | 0.76 | 0.22-2.65 | 1.20 | 0.46-3.15 | | 1.27 | 0.33-4.85 | |  | 0.82 | 0.59-1.13 | 1.37 | 0.63-3.01 |  | | |
| T/T | 1.49 | 0.21-10.42 | 1.45 | 0.52-4.07 | | 4.83 | 0.60-38.89 | |  | 1.02 | 0.63-1.63 | 0.53 | 0.06-4.81 |  | | |
| ACE |  |  |  |  | |  |  | |  |  |  |  |  |  |  |  |
| I/I | 1.00 | reference | 2.14 | 0.58-7.88 | | 2.49 | 0.46-13.31 | | 0.072 | 1.00 | reference | 2.22 | 0.85-5.82 | 0.167 | | |
| I/D | 0.70 | 0.13-3.63 | 2.02 | 0.56-7.33 | | 7.34 | 1.57-34.43 | |  | 1.10 | 0.76-1.60 | 1.20 | 0.53-2.72 |  | | |
| D/D | 3.59 | 0.74-17.38 | 1.87 | 0.51-6.91 | | 0.50 | 0.04-5.63 | |  | 0.93 | 0.60-1.42 | 3.88 | 1.17-12.88 |  | | |
|  |  |  |  |  | |  |  | |  |  |  |  |  |  |  |  |

Multivariate logistic regression analysis. P-value of logistic regression adjusted for sex, age and puberal maduration

Figure S1. The histogram of body mass index, body fat percentaje and waist circumference by gender


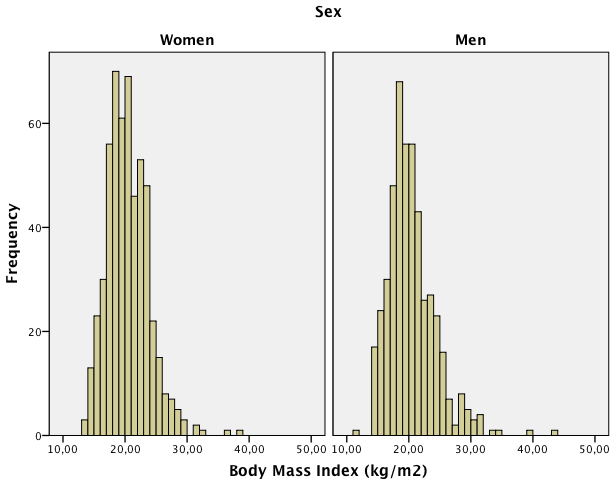


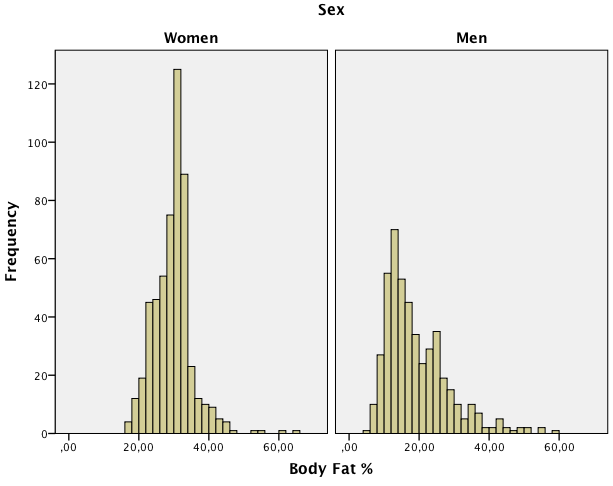


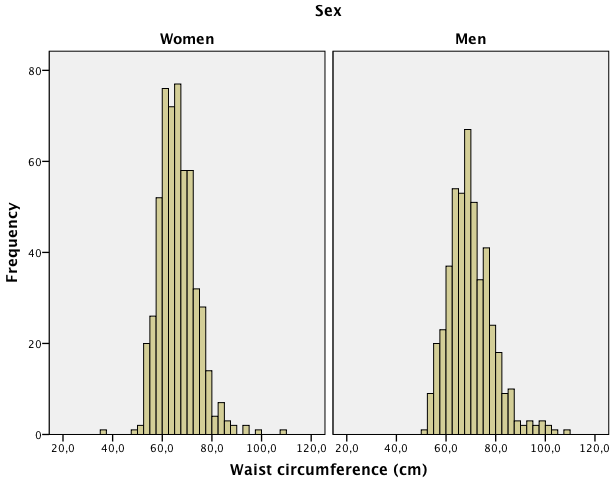

Supplement: Additional file 1: Table S1. — Sequence of primer used to PCR. Table S2. Ancestral informative Markers (AIMs), allelic frequencies and delta (δ) of frequencies in the three ancestral populations (European, African and Amerindian). Table S3. Characteristics of the study population, stratified according to gender. Table S4. Association with anthropometric measures at 10 selected genetic variants on the subset of the samples with available ancestry information. Table S5. Interaction between SNP and socioeconomic and perinatal factors in determining body mass index. Figure S1. The histogram of body mass index, body fat percentage and waist circumference by gender. (DOCX 123 kb) [file 12263_2017_567_MOESM1_ESM.docx]
